# A Perspective on Multiple Waves of Influenza Pandemics

**DOI:** 10.1371/journal.pone.0060343

**Published:** 2013-04-23

**Authors:** Anna Mummert, Howard Weiss, Li-Ping Long, José M. Amigó, Xiu-Feng Wan

**Affiliations:** 1 Department of Mathematics, Marshall University, Huntington, West Virginia, United States of America; 2 School of Mathematics, Georgia Institute of Technology, Atlanta, Georgia, United States of America; 3 Department of Basic Sciences, Mississippi State University, Mississippi State, Mississippi, United States of America; 4 Centro de Investigación Operativa, Universidad Miguel Hernández, Elche, Spain; Massey University, New Zealand

## Abstract

**Background:**

A striking characteristic of the past four influenza pandemic outbreaks in the United States has been the multiple waves of infections. However, the mechanisms responsible for the multiple waves of influenza or other acute infectious diseases are uncertain. Understanding these mechanisms could provide knowledge for health authorities to develop and implement prevention and control strategies.

**Materials and Methods:**

We exhibit five distinct mechanisms, each of which can generate two waves of infections for an acute infectious disease. The first two mechanisms capture changes in virus transmissibility and behavioral changes. The third mechanism involves population heterogeneity (e.g., demography, geography), where each wave spreads through one sub-population. The fourth mechanism is virus mutation which causes delayed susceptibility of individuals. The fifth mechanism is waning immunity. Each mechanism is incorporated into separate mathematical models, and outbreaks are then simulated. We use the models to examine the effects of the initial number of infected individuals (e.g., border control at the beginning of the outbreak) and the timing of and amount of available vaccinations.

**Results:**

Four models, individually or in any combination, reproduce the two waves of the 2009 H1N1 pandemic in the United States, both qualitatively and quantitatively. One model reproduces the two waves only qualitatively. All models indicate that significantly reducing or delaying the initial numbers of infected individuals would have little impact on the attack rate. Instead, this reduction or delay results in a single wave as opposed to two waves. Furthermore, four of these models also indicate that a vaccination program started earlier than October 2009 (when the H1N1 vaccine was initially distributed) could have eliminated the second wave of infection, while more vaccine available starting in October would not have eliminated the second wave.

## Introduction

An influenza pandemic occurs when a new strain of the influenza virus emerges, usually through antigenic shift, for which there is little or no immunity in the human population. The mutation is such that it is able to cause illness in a single individual and then spreads person-to-person worldwide. In the 20th century, the world experienced three influenza pandemics: the 1918 H1N1 "Spanish flu'', the 1957 H2N2 "Asian flu'', and the 1968 H3N2 "Hong Kong flu''. The first influenza pandemic of the 21st century occurred in 2009 and was caused by a swine-origin H1N1 influenza A virus [1].

**Figure 1:**
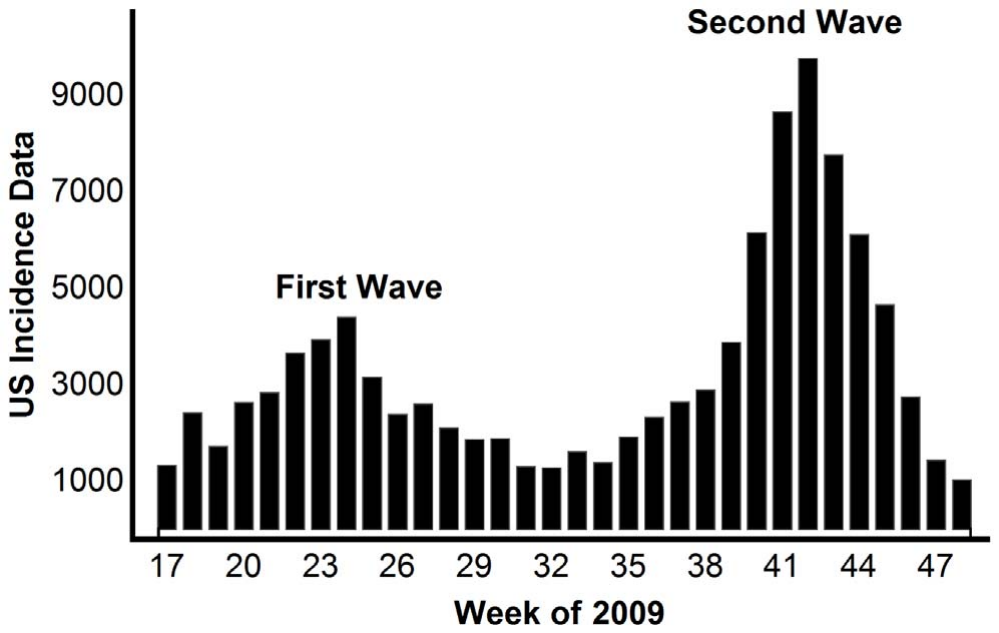
The H1N1 positive tests reported to the CDC in the United States from April 24, 2009 (week 17) to November 27, 2009 (week 48).

During each of these four pandemics, the United States experienced multiple waves of infections, where the numbers of infections and deaths exhibited well-separated temporal peaks with a separation time-scale of months [1]. For example, the first wave of the 2009 pandemic in the United States began in March and peaked in late June and early July. There were markedly fewer cases throughout August, and the second larger wave peaked in late October, early November (Figure 1, Table S1 in File S1). While many countries such as the United States experienced at least two waves of infections during the 2009 pandemic, other countries such as China experienced only a single wave of infection.

The underlying mechanisms leading to single or multiple waves of acute infectious diseases are not well understood. In this paper, we describe and explore several mechanisms that can produce multiple waves.

For several infectious diseases, including influenza, the timing of school vacations is thought to be a mechanism for multiple waves because children have reduced transmission due to far fewer contacts during vacations than when school is in session. Parts of either the summer or winter school breaks occurred during the gaps between the two waves of the past four influenza pandemics in the United States. Evidence supporting this potential mechanism for the second wave of the 2009 US H1N1 pandemic include the surge in hospital outpatient visits for influenza-like illness approximately two weeks after schools re-opened in the fall of 2009 [2]. However, school vacations clearly cannot be the sole cause of multiple waves [2], [3], [4], [5], [6]. For instance, during the 1968 pandemic in the United States, the second wave began in November, two months after school resumed. In addition, children have school vacations every year, while seasonal influenzas have only single waves of infections.

Another possible mechanism for multiple waves is due to rapid mutations of the H1N1 virus. Many RNA viruses, such as influenza, quickly acquire genetic variants through random mutations, some of which lead to non-synonymous mutations in protein sequences. The more genetic variants that there are, the higher the odds are that one of them carries a mutation upon which selection can act. Through random mutation and subsequent selection, RNA viruses evolve into a form better adapted for human-to-human transmission.

The protein sequence analyses for the 2009 H1N1 HA genes showed the average number of mutations increased slightly from April (2.75±0.71, based on the comparison with the A/California/07/2009 strain) to November of 2009 (4.26±1.53). The maximum number of mutations occurred in November 2009 (11 residues when compared with A/California/07/2009, versus two residues when compared with the major strain circulating in April 2009). Virus diversity, which we introduce in the next section, quantifies the extent of the mutations.

Some of these mutations occurred in the receptor binding sites of HA genes [7], [8] and in other segments of the virus [9]. Some mutations first appeared after the first wave ended (e.g., HA-S220T NA-N248D in Japan) [10]. As the pandemic progressed, the number of mutations at the receptor binding site position 222 increased around the world [7], [11], [12], [13], [14]. The mutation D222G/N was observed in 90% of the blood samples of the A/H1N1/2009 viremia cases [8], [15], [16], [17], [18]. The D222G mutation has been shown to increase virulence and to increase the virus transmissibility in animal models, as well in human airway epithelial cell lines [19], [20], [21], [22]. Other mutations in the receptor binding sites of the HA gene [23], [24], and other mutations at other segments (e.g., PA, PB1-F2, PB2, NP, and NS1) have been found in clinical isolates and have been shown to increase the replication efficiency and pathogenesis *in vitro* in animal models. Increased virulence and pathogenesis of such mutations is a mechanism that may lead to a second wave of infection.

In this study, five mathematical models are formulated to explore complementary mechanisms which can produce two waves of acute infections. Four reproduce the two waves of the 2009 H1N1 influenza pandemic, quantitatively. The other reproduces the waves qualitatively. The first two mechanisms capture changing contact rates and changing (or evolving) virus transmissibility. In these models, the changes manifest as a time varying transmission rate. The transmission rate is the per capita rate of infection given contact, and depends in a highly complex way on both the contact rate between susceptible and infected individuals and the transmissibility of the infection. The first mechanism uses a periodic transmission rate to capture the seasonal contact rate. The second mechanism incorporates all sources of variability in contacts and transmissibility into the time-dependent transmission rate.

The third mechanism incorporates a heterogeneous population with individuals split into two weakly interacting sub-populations. The split could be based on demographics, geography, or variations in immunity, among others. The two populations only very weakly interact, that is, the transmission between the two groups is low. Two waves appear with each sub-population experiencing only one wave of infection.

The fourth mechanism is virus mutation which causes delayed susceptibility of some individuals. As the 2009 H1N1 pandemic progressed in the United States, the virus mutated rapidly and created new viable quasispecies. We hypothesize that some of these mutations increased the transmissibility of the influenza virus and that this increased viral transmissibility caused some individuals who were not able to be infected during the first wave to become infected during the second wave.

The fifth mechanism is waning immunity, where recovered individuals lose immunity to the influenza virus at which point they again become susceptible to infection. The second wave appears because some individuals become infected for a second time.

The five mechanisms are incorporated into appropriate extensions of the standard (Susceptible-Exposed-Infected-Removed) transmission model [47]. Models 1–4 reproduce the two waves during the 2009 pandemic in the United States, qualitatively and quantitatively. Model 5 reproduces the two waves qualitatively.

Countries such as China implemented border control strategies and experienced only a single wave of infections. We implement border control in each model to determine whether the US could also have experienced one wave with border control. In addition, the US vaccination production and distribution data is incorporated into the models to test the effect of increasing vaccine availability and altering the timing of the vaccination effort.

## Results

### Models 1 and 2: SEIR models with variable transmission rate

Models 1 and 2 explore the ability of a variable transmission rate to generate the multiple waves of infection in a homogenous population. To do this we use a time-dependent, or variable, transmission rate β_t_ in a classical SEIR compartment transmission model (see Supplementary Information in File S1, equations (S1)–(S4)). We provide two strikingly different transmission rates, each of which can reproduce the two waves. Model 1 exploits seasonality. We demonstrate that the school year hypothesis of a higher transmission rate during the school year and a lower rate during the summer and school vacations can generate the two waves. Clearly, this type of seasonality alone provides no insight into why two waves occur only during pandemic years and not every year; there must be additional factors driving the pandemic waves. Therefore, we provide Model 2, also with a variable transmission rate that captures the intricate interplay between the contact rate and transmissibility of the infection.

### Model 1: Periodic transmission rate

Many experts believe that contacts between school age children play a significant role in influenza transmission and in producing pandemic waves. The contact rate is thought to be significantly higher for school age children during school terms than during school vacations [2], [3], [4], [5], [6]. The presumption is that the first wave begins in the winter or early spring when school is in session and wanes when the children have significantly reduced contact during their summer breaks. The second wave begins soon after school resumes in late summer or early fall, when the contact rate increases.

**Figure 2:**
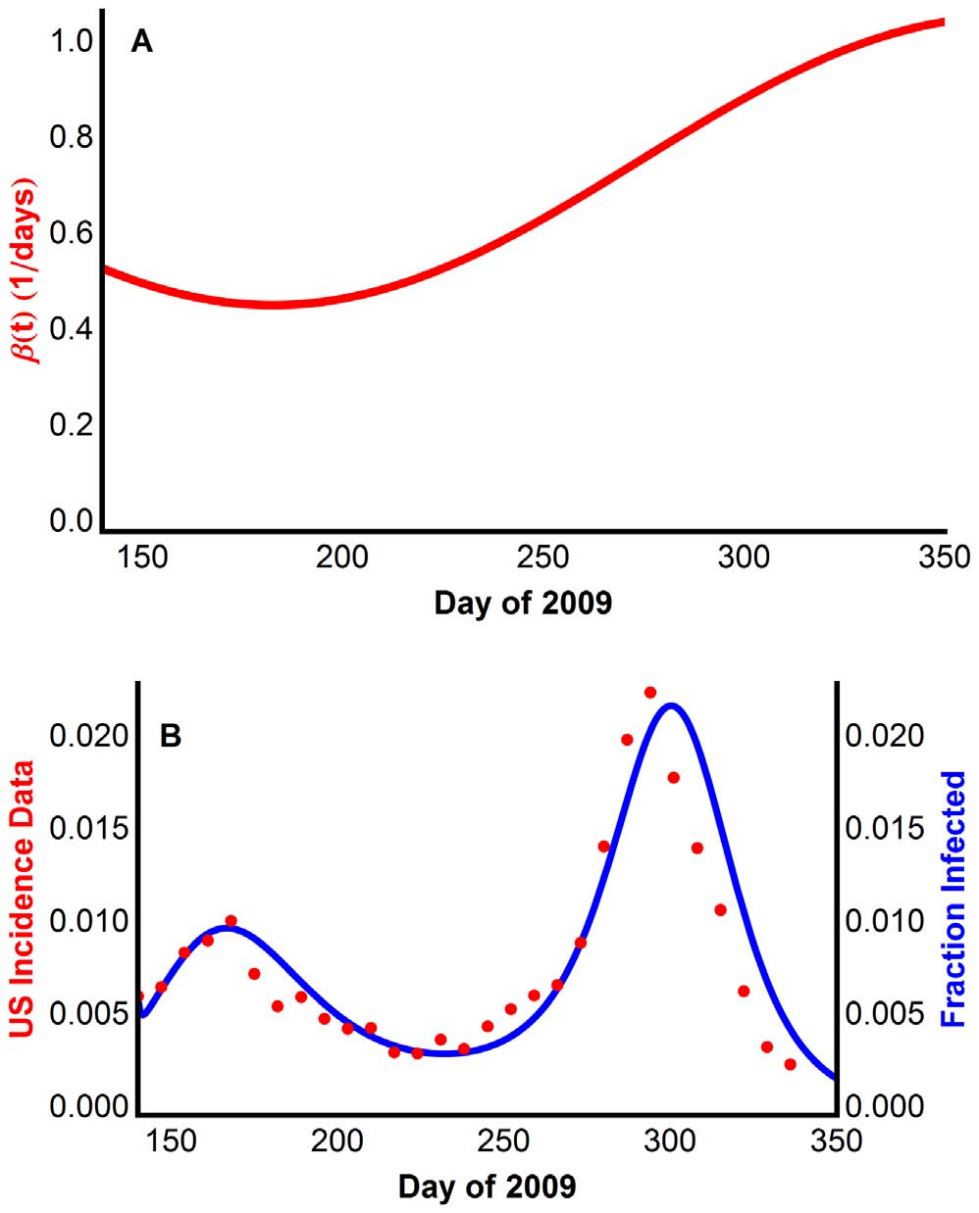
Reproducing multiple waves using Model 1 with periodic time-dependent transmission rate, β_t_. ****(A) The periodic transmission rate, which is low during the summer break and higher when school is in session. Summer is approximately June and July (weeks 23–31 and days 161–217). (B) The model generated disease prevalence, compared with the CDC data scaled for underreporting.

To capture the seasonality of school contacts, the transmission rate β_t_ is set to be the periodic function (with period one year)
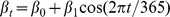
(Figure 2A), where there is higher transmission when students are in school, and lower transmission over the summer months.

The resulting model was simulated using parameters from Table S9 in File S1. Figure 2B shows the output of Model 1 superimposed on the derived CDC incidence data for the United States in 2009. The model clearly captures the two waves of infection.

### Model 2: Derived time-dependent transmission rate

The transmission rate in this model captures all sources of variability of the contact rate between susceptible and infected individuals and the virus transmissibility. This variability could occur because of public health interventions, seasonality, or the evolution of the virus, among other factors.

The transmission rate β_t_ is determined using a new algorithm [25], [26], [27] that ensures the output *I*(*t*) of the model *perfectly* fits a smooth interpolation of the reported cases by the Centers for Disease Prevention and Control (CDC)[28]. There is no error between the model output of *I*(*t*) and the data, and only the initial value β_0_ needs to be specified.

**Figure 3:**
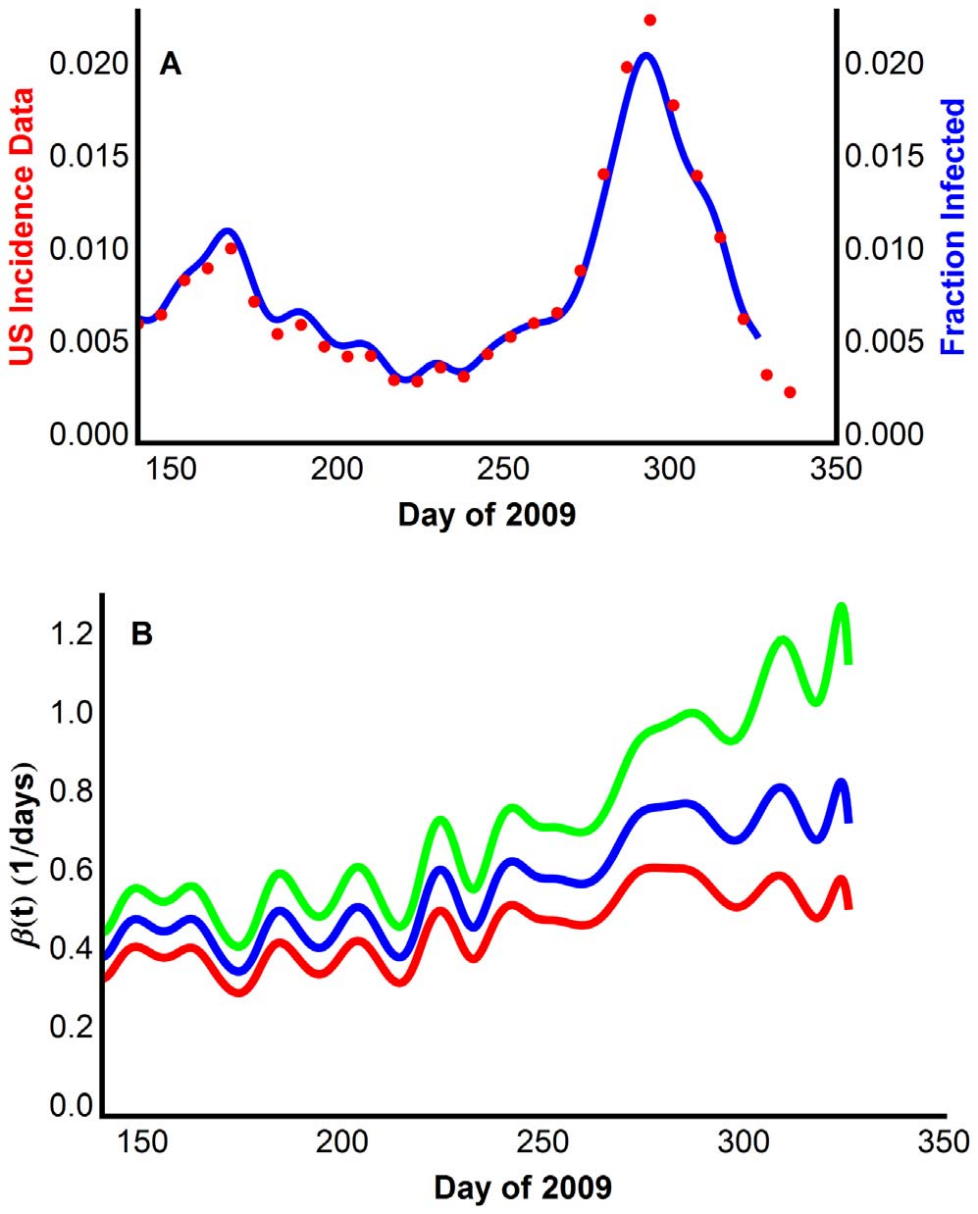
Reproducing multiple waves using Model 2 with extracted transmission rate, βt. (A) The model generated disease prevalence generated for the entire range 0.54 ≤β_0_≤1.03, compared with the CDC data scaled for underreporting. (B) The extracted transmission rates recovered for three β_0_ values. Top green curve β_0_ = 0.73; middle blue curve β_0_ = 0.63; bottom red curve β_0_ = 0.54.

The resulting model was simulated using the parameters in Table S10 in File S1. Figure 3A shows the output of Model 2 superimposed on the derived CDC incidence data for the United States in 2009. Recall that the near perfect agreement was ensured by our choice of β_t_. Errors in the simulation are due to the accumulation of small numerical errors.

Figure 3A was generated using β_0_ = 0.63, however any β_0_ in the range 0.54≤β_0_≤1.03 will produce the same model output. Any β_0_ outside of this range produces unrealistic outputs. Since β_t_ depends on β_0_ in a complicated way, in Figure 3B we show representative graphs of β_t_ across a range of values for β_0_.

In conclusion, Model 2 reproduces the data, and thus the two waves of infection. The mechanism of Model 2 is the construction of a time dependent transmission rate β_t_, capturing all of the fluxuations in transmission due to changing contact rates, virus transmissibility, environment, and so on. The resulting transmission function indicates that the second wave cannot be explained as simply a drop in contacts due to school closures. A drop in contact rate due to school closures would appear as a corresponding drop in β_t_. However, no such drop is seen in the transmission function during the summer months.

### Model 3: Two weakly interacting sub-populations

For this model, we hypothesize that the population is split into two groups with minimal interaction. Each group experiences one wave of infection resulting in two waves for the entire population.

In Model 3, the total population is split into two sub-populations; population 1 is 22% of the total and the rest are in population 2. The SEIR model (Supplementary Information equations (S1)–(S4) in File S1) is extended to eight equations, one S, E, I, and R equation for each sub-population. The sub-populations weakly interact, meaning that the transmission rate between populations is small. This model has three constant transmission rates: β_1_ for the transmission among sub-population 1, β_2_ for the transmission among sub-population 2, and β_3_ for the transmission between the two sub-populations. This model assumes that only sub-population 1 is initially infected and sub-population 2 does not acquire the infection until some time during the summer at which point some infected individuals appear (perhaps arrive home from traveling) who are infected.

**Figure 4:**
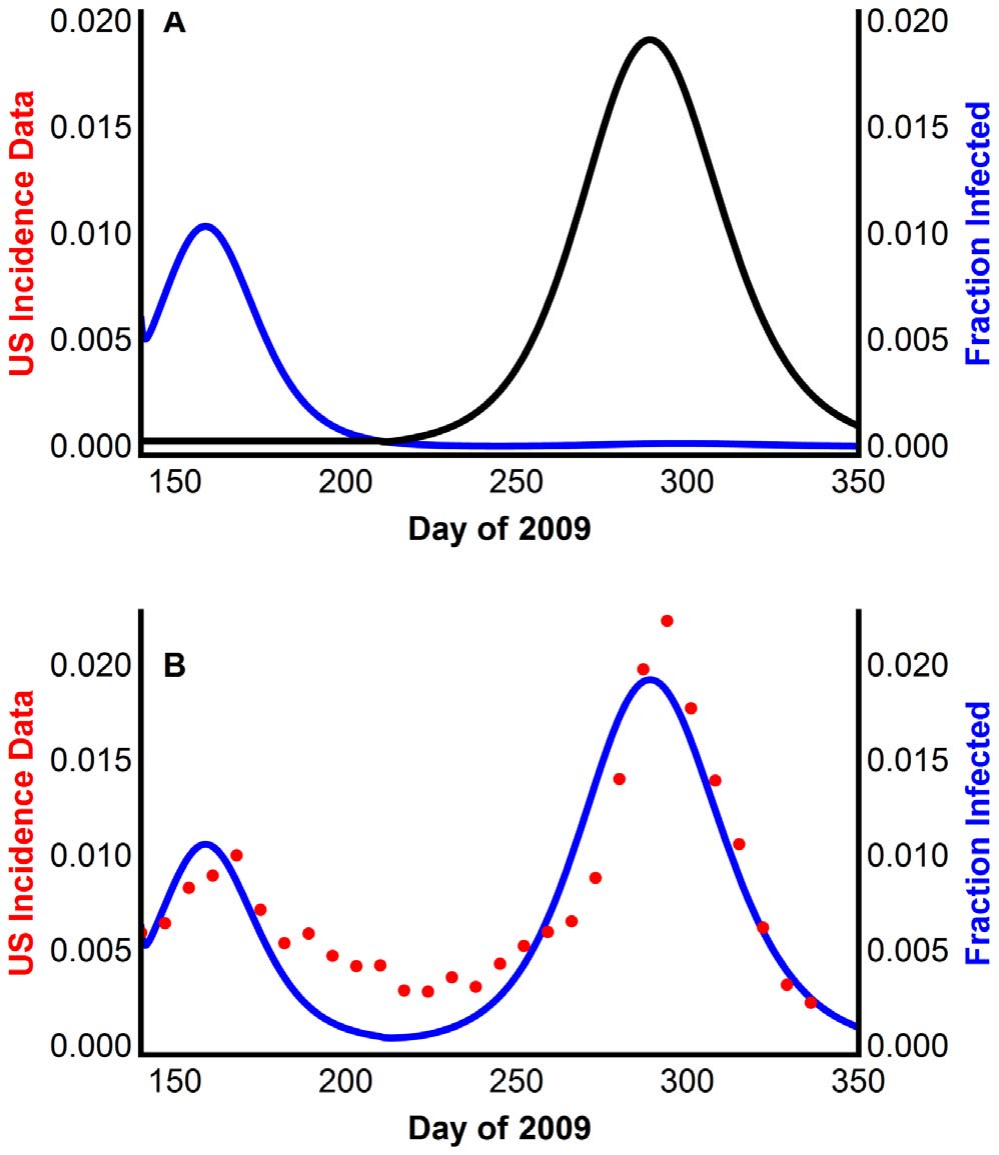
Reproducing multiple waves using Model 3 with two weakly interacting sub-populations. (A) Infection wave of sub-population 1 (blue) and infection wave of sub-population 2 (black). (B) The model generated disease prevalence compared with the CDC data scaled for underreporting.

Model 3 was simulated using the parameters in Table S11 in File S1. Model 3 reproduced the two waves qualitatively (Figure 4B), with each sub-population experiencing only one wave (Figure 4A). Its attack rate is just below that of the 2009 pandemic H1N1 (20.08% compared with the CDC estimate of 25%). These simulations verify that two weakly interacting populations can experience two waves.

### Model 4: Virus mutation and delayed susceptibility

During the course of the 2009 influenza pandemic, the genetic diversity of the virus in both the United States and around the world increased significantly. For this model, we hypothesize that the increase in genetic diversity results in strains with higher transmissibility in the human population. This hypothesis supports the notion that some individuals who were not susceptible to the virus during the first wave become susceptible when new strains with greater transmissibility emerge as the pandemic progresses.

We incorporate this idea into the standard SEIR model (with constant transmission rate *β*) by including a reserved class *N* consisting of individuals who are initially non-susceptible (see equations (S5)–(S9) in the Supplementary Information, File S1). These individuals become susceptible as the viral genetic diversity increases at a rate that is proportional to the time-dependent genetic diversity *d*(t).

**Figure 5:**
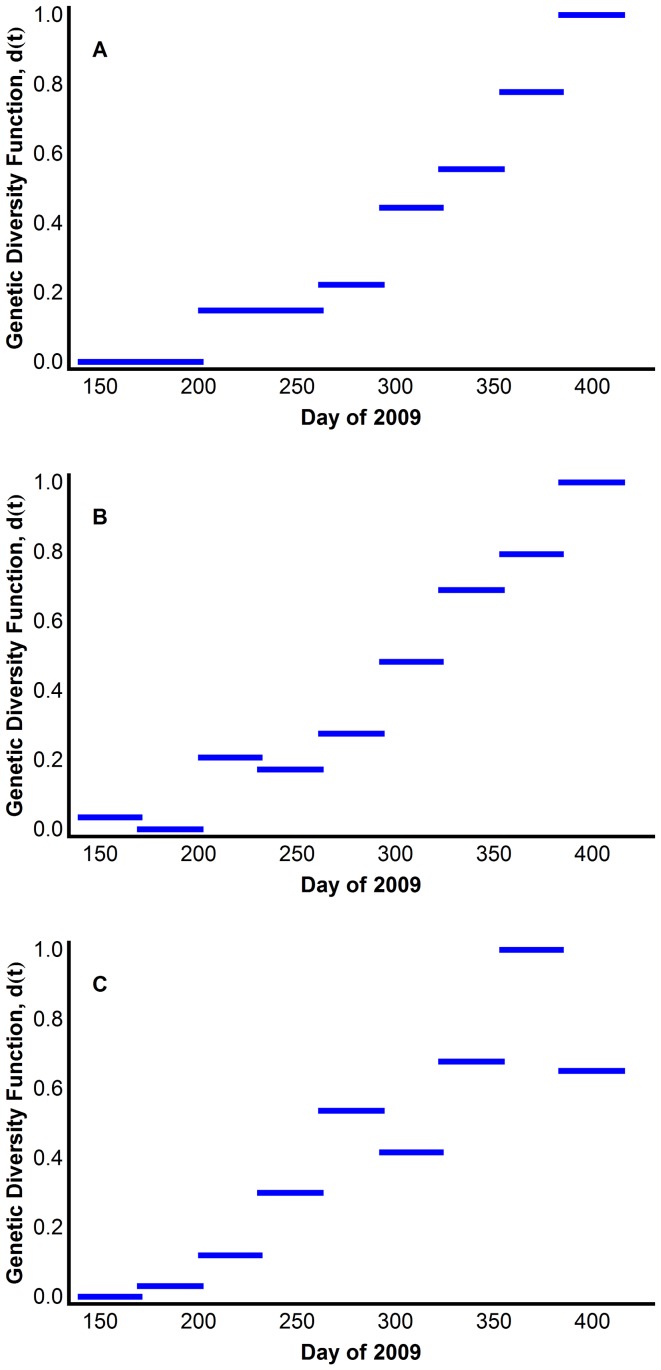
Dynamics of genetic diversity of the HA gene of 2009 H1N1 pandemic virus in the United States. Genetic diversity is defined as the mean of pairwise genetic distances among the genomes of the viruses isolated in one month. The distances were normalized to attain values between 0 and 1. Two genetic distances were measured: (A) p-distance, (B) patristic distance, (C) CCV distance. The genetic diversity of NA gene and other internal genes are shown in Tables S1, S2, S3, S4, S5, S6, and S7 in File S1.

Genetic diversity of virus quasispecies is defined as the mean of pairwise genetic distances among the genomes of the viruses isolated in one month. In bioinformatics there are various measures of genetic diversity. Here, genetic diversity was measured using three distances: the *p-distance*, the *patristic*, and the *CCV distance* between nucleotide sequences. These distances were normalized to attain values between 0 and 1, and the genetic diversity function was generated assuming that the diversity was constant throughout the entire month. In the United States data both of these genetic diversities increased slowly during the first wave and dramatically during the second (Figure 5, Tables S2, S3, S4, S5, S6, S7, and S8 in File S1).

**Figure 6:**
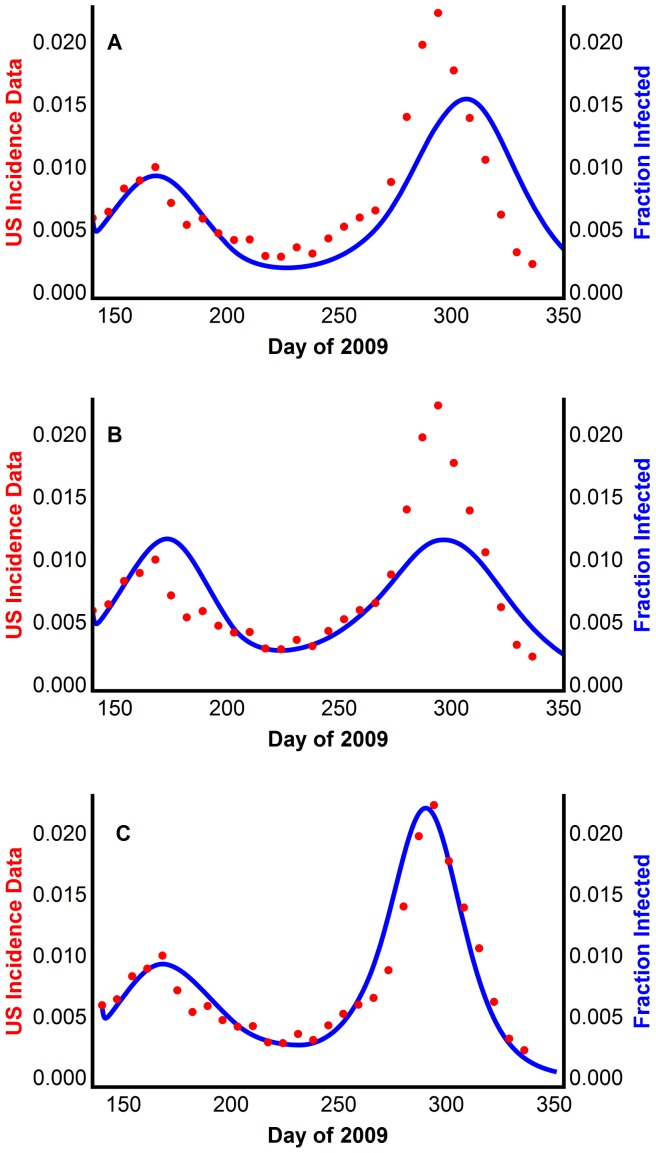
Reproducing multiple waves using Model 4 with virus mutation causing delayed susceptibility of some individuals. The model generated disease prevalence using the (A) p-distance, with *γ*  = 0.11, (B) patristic distance, with *γ* = 0.09, (C) CCV distance, with *γ* = 0.08, compared with the CDC data scaled for underreporting.

Model 4 reproduced the two waves using all three distances (Figure 6), using the parameters in Table S12 in File S1. Here, we construct the genetic diversity function as a step function of order 0. However, Model 4 will also reproduce the second wave using linear, quadratic, etc., interpolations of the monthly distance data. Thus, the model is robust in terms of the distance measure used and the way that these distance measurements are combined to determine the diversity function *d*(*t*).

In conclusion, Model 4 qualitatively reproduces the US incidence data and quantitatively reproduces the attack rate for the 2009 pandemic H1N1. This model lends support to the supposition that virus mutations can drive the second wave through the addition of susceptible individuals who were not susceptible to previous virus quasispecies. The unknown factor of Model 4 is the initial fraction of reserve non-susceptible individuals.

Here the value *N*(*0*) = 0.35 was chosen so that the simulation recreated the data.

### Model 5: Waning immunity

A fifth mechanism is known to produce two waves of infection – waning immunity [8],[36]. We incorporate waning immunity to the H1N1 virus with an SEIR model where individuals in the removed class lose immunity at a constant rate and rejoin the susceptible class (see Supplementary Information equations (S10)–(S13), in File S1).

**Figure 7:**
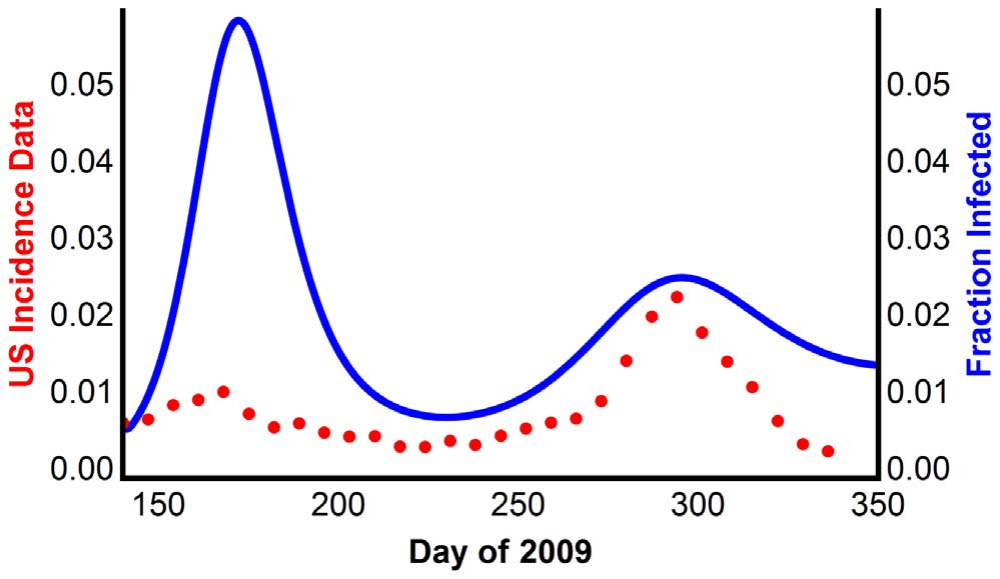
Reproducing multiple waves using Model 5 with waning immunity. The model generated disease prevalence, compared with the CDC data scaled for underreporting.

This model was simulated using the parameters in Table S13 in File S1. It qualitatively reproduces the two waves, but always (necessarily) with a smaller amplitude second wave (Figure 7).

### The effect of initial infections on the second wave

The models were used to investigate the consequences of a smaller number of initially infected individuals in the United States and to identify factors that may affect the on-set of waves.

**Figure 8:**
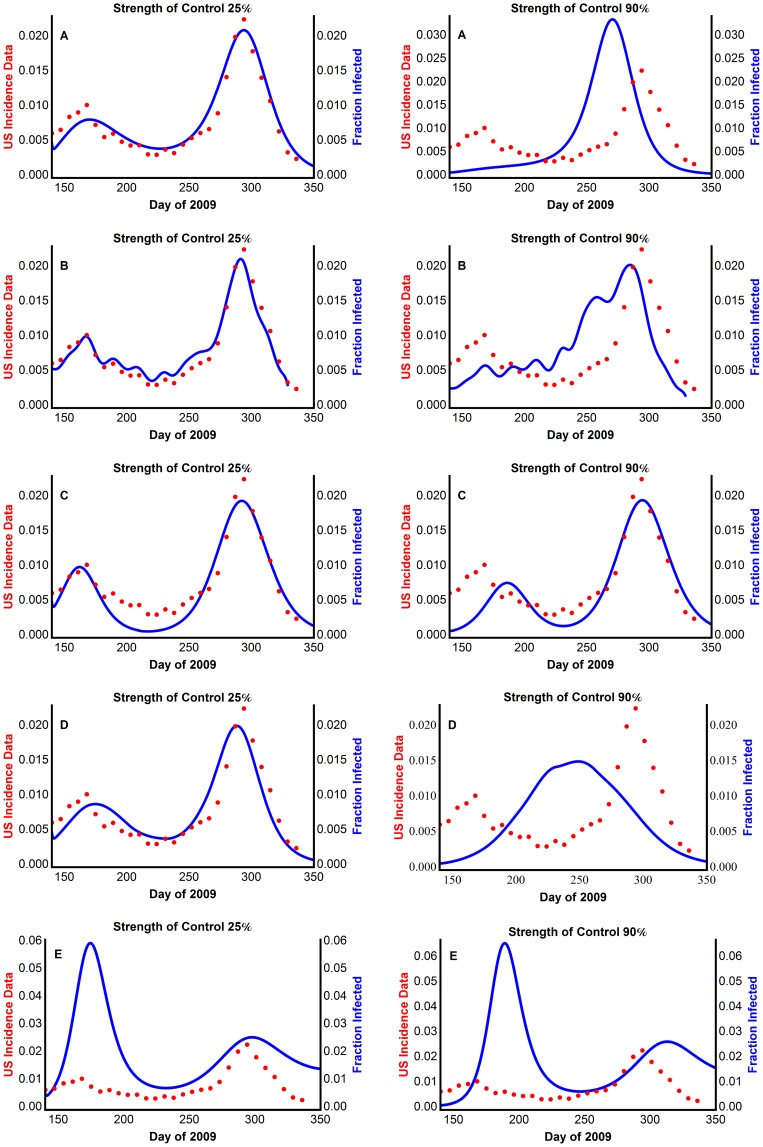
Effects of reduced and delayed initial infected individuals on influenza prevalence using (A) Model 1 with periodic transmission rate (B) Model 2 with extracted transmission rate, β_t_ (C) Model 3 with two weakly interacting sub-populations (D) Model 4 with virus mutation causing delayed susceptibility of some individuals (E) Model 5 with waning immunity. The model generated disease prevalence with the control strength indicated, compared with the CDC data scaled for underreporting, where 25% strength of control means that the initial fraction of infected individuals is 75% of the adjusted reported fraction.

We assumed that an early intervention, e.g., border control, would have reduced the initial number of imported cases from Mexico. We included this in our models by reducing the initial fraction of infected individuals, *I*(0). For Model 3, we reduce the number of individuals of both sub-population 1 and 2. The simulation outputs (Figure 8) illustrate the result of two different intervention levels. The first plot for each model shows the resulting number of infected individuals, assuming that interventions reduced the initial number of infected individuals by 25% of the reported value adjusted for underreporting, while the second plot corresponds to a simulation output that reduced the number of individuals initially infected by 90%.

A value, called the attack rate (*I_total_*), was computed for all models. The attack rate is the total fraction of individuals who become infected during the disease outbreak. With no intervention, the attack rate is *I_total_* = 0.2457, 0.2258, 0.2008, and 0.2307 for Models 1, 2, 3, and 4, respectively. The attack rate barely changes with the addition of interventions, even strong interventions. For example, with a 90% effective intervention, the attack rate is *I_total_* = 0.2379, 0.2324, 0.1978, and 0.2132 for Models 1, 2, 3, and 4, respectively. In addition, in the simulations of Models 1, 2, and 4, interventions of over 80% result in a loss of the second wave. For Model 5, individuals may lose immunity to the disease and become infected a second time. Thus the notion of attack rate is not as well defined. We compute instead the total number of individuals who become infected once throughout the outbreak as 0.7395, and 0.7361 with 90% effective intervention.

### The effect of vaccinations on the second wave

**Figure 9:**
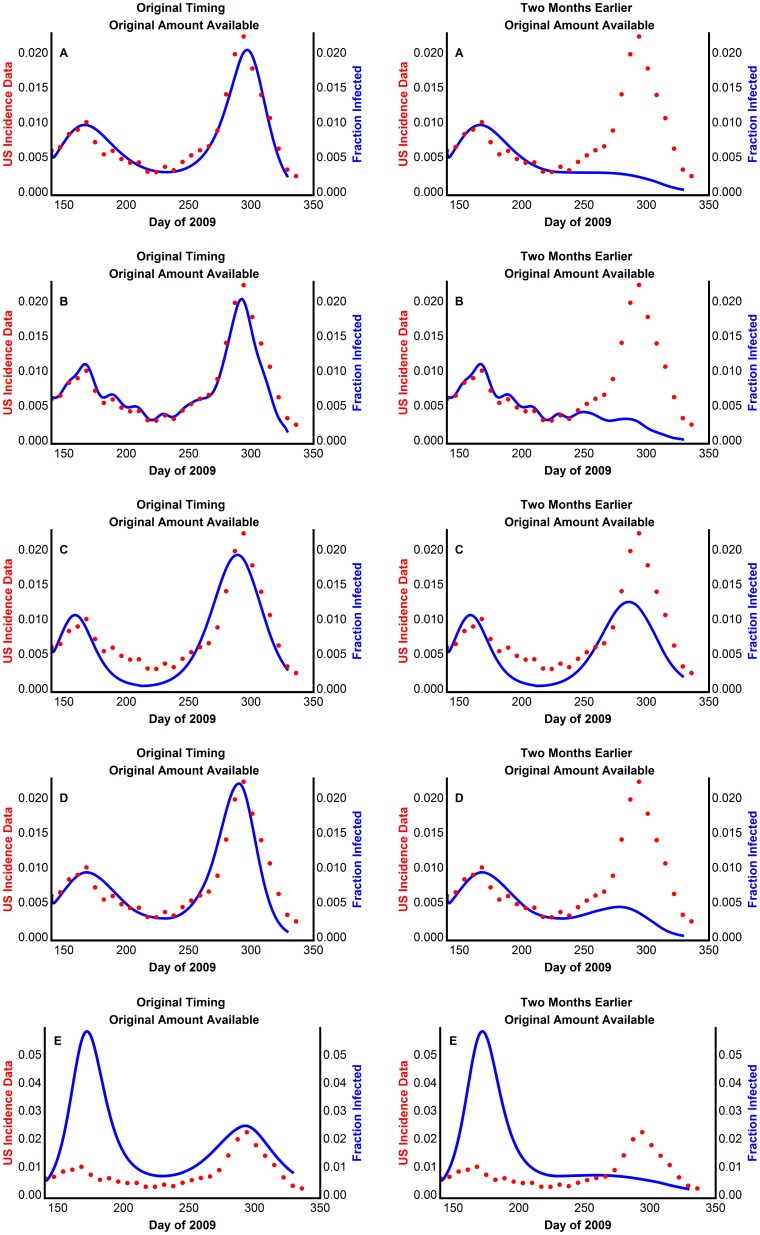
Effects of timing and amount of available vaccine on influenza prevalence using (A) Model 1 with periodic transmission rate (B) Model 2 with extracted transmission rate, β_t_ (C) Model 3 with two weakly interacting sub-populations (D) Model 4 with virus mutation causing delayed susceptibility of some individuals (E) Model 5 with waning immunity. The model generated disease prevalence with the vaccination strategy indicated, compared with the CDC data scaled for underreporting. Here original timing begins vaccination on October 14, 2009, and one month earlier begins vaccination thirty days prior; original amount available is the daily amount of shipped vaccine with 90% efficacy, and twice as much available is twice the shipped amount with 90% efficacy.

The models were used to investigate the consequences of a vaccination program started earlier or with an increased availability of vaccine in the United States on the outbreak progression. We assumed that vaccination would have reduced the number of susceptible (and reserve non-susceptible) individuals by the number of available vaccines, with a vaccine efficacy of 90% [29]. We have accounted for individuals receiving the vaccine according to the dates and amounts of distributed vaccine, along with a two week delay [30]. During the delay the immune system is generating antibodies and is still vulnerable to infection. We investigated both the scenarios of earlier available vaccine (0–5 months earlier) and the scenarios of more available vaccine (1–5 times as much available). Figure 9 illustrates the result of different vaccination efforts for the models.

Earlier vaccinations can both eliminate the second wave of infections and significantly reduce the attack rate. Simulations indicate that vaccinations must be distributed approximately 2 months prior to the actual distribution dates to eliminate the second wave in Models 1, 2, 4, and 5. A larger availability of vaccine can only reduce the attack rate when combined with earlier vaccination. For example, with the original amount of vaccine distributed in October 2009, the attack rate is *I_total_* = 0.2142, 0.2072, 0.1931, and 0.2143 for Models 1, 2, 3, and 4, respectively, while with five times the original amount distributed at the same time, the attack rate is *I_total_* = 0.1788, 0.1845, 0.1689, and 0.1931 for Models 1, 2, 3, and 4, respectively. For Model 5, the total number who are infected once with the original vaccine distribution is 0.6951, while it is 0.6670 with five times the original amount distributed with the original timing.

Table 1 provides a summary of the five models, including the number of peaks of infection resulting from border control and vaccination, the attack rate with border control, and a reference for the model, if available.

**Table 1:** Summary of border control and vaccination on multiple waves of 2009 H1N1 influenza pandemics.

Model	Description	Result of Border Control (90%)	Attack Rate with Border Control (CDC est 25% with no Border Control)	Result of Vaccinating Two Months Earlier	Reference
1	Periodic transmission rate	One wave	24%	One wave	[46]
2	Extracted transmission rate	One wave	23%	One wave	
3	Two weakly interacting populations	One wave (BC 99.9%)	20%	Two waves	[47]
4	Genetic diversity of flu quasispecies	One wave	23%	One wave	
*5*	Waning immunity	Two waves	73%	One wave	[8]

## Discussion

We study five mechanisms that can produce two waves of infection, and may explain the two waves experienced in the United States during the 2009 pandemic H1N1. These mechanisms are implemented and explored in mathematical models. Four models perform well, recreating the two waves (Figures 2, 3, 5 and 7) qualitatively and quantitatively, while one recreates the two waves only qualitatively. The two waves can be reproduced using the models individually or in any combination.

Two novel mechanisms (Models 2 and 4) are proposed. Models 1 and 2 incorporate a variable transmission rate, which was assumed constant in Models 3, 4 and 5. Model 4 explicitly incorporates the genetic diversity of the influenza quasispecies. We deem these models (2 and 4) complementary since changes in the transmission rate and transmission patterns among sub-populations could have been due, in part, to the emergence of more transmissible H1N1 mutants, which increases the genetic diversity of the quasispecies.

Model 1 is an SEIR component model of transmission with a time-dependent transmission rate reflecting the contact rate of school age children. It produces two waves with realistic parameter values, but provides no insight into why two waves of infection appear only with pandemics and not every year.

Model 2 is an SEIR model of transmission with a time-dependent transmission rate. Such a time-dependent transmission rate can account for temporal changes in virulence, seasonality, and contact rates, among others. The transmission rate is obtained from an algorithm that guarantees that the model output perfectly agrees with the infection data, even if the assumptions of the model do not apply. Thus one must be careful applying this algorithm and not over fit the data. We do not use our models to predict numbers of infections; we use them to exhibit possible mechanisms for two waves. In addition, we believe that the assumptions of a time-dependent transmission rate along with the structure of an SEIR model are reasonable for influenza, thus the potential weakness of over fitting is avoided.

The extracted transmission rate for Model 2 seems to be at odds with the common belief that summer affords fewer interactions amongst children [2], [3], [4], [5], [6] and therefore causes a drop in the transmission rate. For all graphs in Figure 3B, there was no significant drop in β_t_ after the school year ended; the transmission rate fluctuated around a nearly constant level in the first wave from mid-May through the end of August. It increased when the fall school term and the second wave began. Although at first surprising, there is no contradiction with a decrease in the number of infected individuals and a (basically) constant transmission rate, as is seen over the summer months. A constant transmission rate implies that the ratio of new infections to the number of susceptible individuals remains constant, which happens over the summer when both the numbers of new infections and of susceptible individuals are decreasing.

From days 150 to 250, the transmission rate β_t_ oscillated with mean period 19.9. These oscillations appear more rapid than the contact rate and environmental factors can account for. We do not see how this oscillation can be a manifestation of reporting conventions. However, these oscillations seem essential to reproduce the reported data. Figure S1A in File S1 shows a 21-day Gaussian filtered (smoothed) β_t_ function, and the corresponding model simulation (Figure S1B in File S1), which is a substantially lower quality fit to the data. All attempts at smoothing the oscillations using different types of filters resulted in a worse fit to the data. Presumably, smoothing the data would eliminate these oscillations, but this goes in a very different direction.

Model 3 consists of two weakly coupled SEIR models of transmission, with a constant transmission rate. We have found that this model is the least robust, since it is the most sensitive to changes in the model parameter values. By construction, the model will always exhibit two waves regardless of the initial number of infected individuals (e.g., with a strong border control).

Model 4 involves virus mutation causing delayed susceptibility of some individuals. This new mechanism posits that as the genetic structure of the influenza virus changed during the pandemic, more of the general population became susceptible to infection. Such a dynamic of transmissibility of influenza A virus has been hypothesized to generate the second wave [31]. Model 4 assumes that the initially non-susceptible individuals become susceptible at a rate proportional to the genetic diversity of the flu quasispecies. The effects of vaccination and border control are considered for Model 4 (Figure 8D, Figure 9D) using the CCV distance, and similar results hold for both the p-distance and the patristic distance.

Model 5 incorporates waning immunity to the influenza virus causing some individuals to become infected again. This model qualitatively reproduces the two waves of the 2009 H1N1 pandemic in the US; however, the first wave will always be larger than the second. In addition, waning immunity requires an unrealistically short period of immunity (six months) to accurately reproduce the timing of the two waves of the 2009 H1N1 pandemic. For these reasons, we believe that this mechanism played at most a minor role in the second wave.

While all combinations of all five models will generate the two waves, it is impossible to determine the actual mechanism(s) from any model. This is a fundamental limitation of mathematical modeling. Models can suggest plausible mechanisms, but they cannot prove them. There is so little data from other countries and other pandemics that any type of statistical investigation trying to narrow down the true explanation is impossible.

Although age structure probably plays a role in flu transmission, our models do not explicitly include such structure and they do not need to. We are able to illustrate that all five mechanisms can reproduce two waves without explicit age structure in the model. We point out that the two populations in Model 3 could be two age groups, though other groups are also possible.

### The transmission rate during the summer school vacation

For all graphs in Figure 3B, there was no significant drop in β_t_ after the school year ended; the transmission rate fluxuated around a nearly constant level in the first wave from mid-May through the end of August. It increased when the fall school term and the second wave began. Although at first surprising, there is no contradiction with a decrease in the number of infected individuals and a (basically) constant transmission rate, as is seen over the summer months. A constant transmission rate implies that the ratio of new infections to the number of susceptible individuals remains constant, which happens over the summer when both the numbers of new infections and of susceptible individuals are decreasing.

### The effects of border control

During the 2009 H1N1 pandemic, countries including China, Japan, Hong Kong, Singapore, and Australia implemented various border control strategies to prevent arriving airline passengers from infecting the countries' citizens [32], [33], [34], [35]. Health authorities screened arriving passengers for flu-like symptoms using thermal scanners. In China, passengers suspected of being infected, along with passengers seated nearby, were placed into quarantine for seven days. China's border control began when the first cases of H1N1 were confirmed in California; China continued screening passengers for more than two months.

There has been debate as to whether the United States should have implemented some form of border control with Mexico after the first novel influenza cases appeared. The results from simulations indicate that strong intervention at the beginning of the outbreak would have had negligible impact on the attack rate in the United States, but it could have resulted in a single wave of infections. Our simulations imply that by significantly decreasing the initial number of infected individuals, the two waves would collapse into a single wave of infections, and the peak number of infections would occur slightly earlier with one wave. This suggests that China's strong border control could be a potential mechanism explaining their one wave of infection.

In practice, the efficacy of such border control measures to reduce transmission is uncertain. These screenings will miss asymptomatic individuals [35], and in particular infected individuals with no fever. However border control has been shown to delay the local community transmission [32], [33], [34], which may allow sufficient time for health interventions, such as vaccinations. On the other hand, border control may only delay transmission and could still result in a second wave, as seen in the simulations.

### The effects of vaccination

The first doses of 2009 H1N1 vaccine were available in the United States in early October 2009, which was just before the peak of the 2009 H1N1 pandemic. Conventional wisdom is that this vaccination had little effect on the course of the disease spread. Though we were unable to find any empirical evidence in the literature, our simulations support this idea. Simulations of all models indicate that the actual vaccine schedule did not significantly reduce the total number of infections and even a significantly larger amount of vaccine available in October would have little effect on the attack rate. However, an earlier vaccination program would significantly decrease the attack rate and can eliminate the second wave of infections. Simulations show that the attack rate would be significantly reduced had the vaccine been distributed two months earlier than it was (with the same availability).

Recall that by design, Model 3 will produce two waves. Extremely strict border control (99.9% control) can prevent the second wave; however starting the vaccination program five months earlier with the original amount of vaccine cannot prevent the second wave.

## Materials and Methods

### Data sets

The epidemiologic dataset of 2009 weekly H1N1 positive tests reported to the CDC was downloaded from the CDC online weekly reports (www.cdc.gov/flu/). Clearly, these weekly numbers are highly affected by the testing efforts. An estimated 25% of the U.S. population was infected with the pandemic H1N1 throughout the two waves [36]. Using this along with the CDC compiled data, the influenza confirmed case data was adjusted to reflect underreporting by a scale factor of 700∶1. The authors are aware that a team of investigators estimated an underreporting scale factor of 79∶1 at the beginning (April through July) of the pandemic [32]. This is not a contradiction, since, very quickly, many physicians and hospitals stopped testing and the underreporting factor skyrocketed. Since we could find so little information about the underreporting rate during the entire pandemic in the U.S., we chose a uniform scaling factor.

The genomic sequence dataset was downloaded from the Influenza Virus Resource Database [37], which was updated on November 16, 2011. This dataset includes 1,502 HA genes of 2009 pH1N1 pandemic influenza viruses (229 were isolated in April of 2009; 273 in May; 247 in June; 74 in July; 46 in August; 119 in September; 195 in October; 214 in November; and 105 in December). All HA genes were fully or nearly fully sequenced, and only a single HA sequence for the same strain was selected.

### Measurement of genetic distance and genetic diversity

The genetic diversity of the influenza virus quasispecies is defined as the mean of the pairwise genetic distances among the genes of the virus. We employ three notions of genetic distance: the p-distance, the patristic distance, and the CCV distance [38], [39]. P-distance measures the proportion of nucleotide sites between two sequences. This is the simplest measure and does not take into consideration multiple substitutions at the same site, substitution rate biases, or differences in the evolutionary rates among sites [40]. The p-distances were measured using the software package MEGAN [41]. The patristic distance encodes the total amount of genetic change that exists between genetic sequences by summing the lengths of branches in the phylogenetic tree between the two viruses. The patristic distances were calculated using PATRISTIC [42] using the maximum likelihood phylogenetic trees constructed by GARLI [43]. CCV is a method for measuring genetic distance based on the probability of strings [Wan et al. 2007], and this method has been shown to be effective in measuring the genetic distance between influenza genes [Wan et al. 2007b; Wan et al. 2008a; Wan et al. 2008b; Long et al. 2012].

### Model parameter selection

Every model parameter value is consistent with the range commonly found in the literature. Within the common ranges, parameters for Models 1–4 were found that quantitatively and qualitatively reproduce the two waves of the 2009 H1N1 pandemic in the US. For Model 5, no parameters were found from the common ranges that reproduce the two waves quantitatively.

The incubation period for influenza can range between one and four days. We follow modeling convention and use a one day incubation period in all models. Adults shed influenza virus for up to 11 days; however, the amount shed is significantly lower after the first 3 to 5 days. Several previous modeling studies of pandemic H1N1 have assumed the infectivity period (1/*v*) is the lower bound of 3 days, as is done in all models here.

The transmission rate *β* (or the initial transmission rate for Models 1 and 2) is chosen to ensure that the basic reproduction number R_0_ is in the allowable range for influenza. The basic reproduction number is the average number of secondary cases caused by one infected individual into an entirely susceptible population. However, it is well known that approximately 20% of the US population had prior immunity to the pandemic influenza. Therefore, it is unreasonable to compute R_0_ assuming that the entire population was susceptible. We use instead the standard definition where it is not assumed that all individuals are initially susceptible. For the 2009 H1N1 influenza, R_0_ is estimated to be in the range 1.2–2.25 [44]. For Model 1, the transmission rate at the start of the outbreak (t = 140) is 0.5267, resulting in an R_0_ of 1.58. For Model 2, simulations restrict β_0_ to the range 0.54 to 1.03. Figure 3 shows β_0_ in the range 0.54 to 0.74, which gives a basic reproduction number in the range 1.39 to 1.88. For Model 3, at the beginning of the infection β_1_ = 2.1, which gives a basic reproduction number of R_0_ = 1.386, and for the second wave, β_2_ = 0.565, which gives a basic reproduction number of R_0_ = 1.322. For Model 4, β = 0.9, and so the basic reproduction number is R_0_ = 1.20. For Model 5, β = 0.63, and so the basic reproduction number is R_0_ = 1.89.

For Models 1, 3, 4, and 5, the initial fraction of infected individuals, *I*(*0*) (I_1_(0) for Model 3) is set by the US incidence data and the initial fraction of exposed individuals *E*(*0*) is set to be 0. For Models 1, 4 and 5, the initial fraction of removed individuals *R*(*0*) is set to 0.2, corresponding to elderly individuals with acquired immunity [45]; in Model 3, this value is set to 0. For Model 3, the infection in sub-population 2 begins at day t = 210 and I_2_(210) is the US incidence data scaled by 0.06.

For Model 2, the initial values *S*(*0*), *E*(*0*), *I*(*0*), *and R*(*0*) are determined by the algorithm which computes the time-dependent transmission rate β_t_. Therefore the choice of β_0_ determines all four of the initial values. For β_0_ = 0.63 the initial conditions agree reasonably well with the initial conditions of the other models.

For Model 4, N(0) was chosen to ensure that the basic reproduction rate R_0_ was within the known bounds. With β_0_ = 0.9, the value of N(0) can range from 0 to 0.35. In addition, model simulations show that to generate two waves, the value of N(0) must be larger than 0.25. With the chosen value of N(0) = 0.35 the value of S(0) is forced to be 0.44.

## Supporting Information

File S1 Supplementary Equations, Tables, and Figures.
(DOC)
