# Supplementary material for: A Perspective on Multiple Waves of Influenza Pandemics
Source: PLoS One. 2013 Apr 23;8(4):e60343. doi: 10.1371/journal.pone.0060343 (PMC3634039; doi:10.1371/journal.pone.0060343)
Supplement: File S1 — Supplementary Equations, Tables, and Figures. (DOC) [file pone.0060343.s001.doc]

**Supplementary Information**

## A perspective on multiple waves of influenza pandemics

Anna Mummert^1^, Howard Weiss^2^, Li-Ping Long^3^, José M. Amigó^4^, and Xiu-Feng Wan^3^,*

^1^Department of Mathematics, Marshall University, Huntington, WV, the United States;

^2^School of Mathematics, Georgia Institute of Technology, Atlanta, GA, the United States;

^3^Department of Basic Sciences, Mississippi State University, Mississippi State, MS, the United States;

^4^Centro de Investigación Operativa, Universidad Miguel Hernández, Elche, Spain

*Correspondence: Dr. Xiu-Feng Wan, Mississippi State University, E-mail: [wan@cvm.msstate.edu](mailto:wan@cvm.msstate.edu) or [wanhenry@yahoo.com](mailto:wanhenry@yahoo.com). Phone: +1 (662) 325-3559.

**Methods**

***Equations for Models 1 and 2***

$dS/dt=-\beta_{t}SI$ (S1)

$dE/dt=\beta_{t}SI-\alpha E$ (S2)

$dI/dt=\alpha E-\upsilon I$ (S3)

$dR/dt=\upsilon I$ (S4)

where *S*(*t*), *E*(*t*), *I*(*t*), and *R*(*t*) are the fractions of susceptible, exposed, infected, and removed individuals at time *t*, 1/*v* is the infectious period, and 1/*α* is the latency period for the disease.

***Equations for Model 4***

$dS/dt=-\beta SI+\gamma d(t)N$ (S5)

$dE/dt=\beta SI-\alpha E$ (S6)

$dI/dt=\alpha E-\upsilon I$ (S7)

$dR/dt=\upsilon I$ (S8)

$dN/dt=-\gamma d(t)N$ (S9)

where *S*(*t*), *E*(*t*), *I*(*t*), *R*(*t*), and *N*(*t*) are the fractions of susceptible, exposed, infected, removed, and reserve non-susceptible individuals at time *t*, *β* is the (constant) transmission rate, 1/*v* is the infectious period, 1/α is the latency period for the disease and *𝛾* is the scaling term on the genetic diversity function *d(t)*. The rate at which the fraction of individuals who are initially not susceptible become susceptible is 1/(*𝛾d(t))* per day.

***Equations for Model 5***

$dS/dt=-\beta SI+\phi R$ (S10)

$dE/dt=\beta SI-\alpha E$ (S11)

$dI/dt=\alpha E-\upsilon I$ (S12)

$dR/dt=\upsilon I-\phi R$ (S13)

where *S*(*t*), *E*(*t*), *I*(*t*), and *R*(*t*) are the fractions of susceptible, exposed, infected, and removed individuals at time *t*, *β* is the (constant) transmission rate, 1/*v* is the infectious period, 1/α is the latency period for the disease, and 1/*φ* is the duration of immunity.

**Table S1.** Weekly data from CDC on the laboratory confirmed 2009 pandemic H1N1 cases in the United States.

| Year of 2009 | | Year of 2010 | |
| --- | --- | --- | --- |
| Week Number | Confirmed Cases | Week Number | Confirmed Cases |
| 17 | 1,300 | 1 | 266 |
| 18 | 2,392 | 2 | 261 |
| 19 | 1,697 | 3 | 317 |
| 20 | 2,600 | 4 | 268 |
| 21 | 2,810 | 5 | 290 |
| 22 | 3,628 | 6 | 238 |
| 23 | 3,903 | 7 | 260 |
| 24 | 4,369 | 8 | 316 |
| 25 | 3,128 | 9 | 338 |
| 26 | 2,359 | 10 | 359 |
| 27 | 2,574 |  |  |
| 28 | 2,069 |  |  |
| 29 | 1,831 |  |  |
| 30 | 1,852 |  |  |
| 31 | 1,273 |  |  |
| 32 | 1,250 |  |  |
| 33 | 1,578 |  |  |
| 34 | 1,356 |  |  |
| 35 | 1,884 |  |  |
| 36 | 2,294 |  |  |
| 37 | 2,617 |  |  |
| 38 | 2,859 |  |  |
| 39 | 3,847 |  |  |
| 40 | 6,121 |  |  |
| 41 | 8,628 |  |  |
| 42 | 9,735 |  |  |
| 43 | 7,738 |  |  |
| 44 | 6,083 |  |  |
| 45 | 4,629 |  |  |
| 46 | 2,715 |  |  |
| 47 | 1,408 |  |  |
| 48 | 997 |  |  |
| 49 | 610 |  |  |
| 50 | 480 |  |  |
| 51 | 251 |  |  |
| 52 | 285 |  |  |

**Table S2.** Patristic distance and p distance for PB2 genes of 2009 pandemic H1N1 influenza A viruses from April of 2009 to December of 2009.

| Month | **patristic distance** | | | | | |
| --- | --- | --- | --- | --- | --- | --- |
|  | **Average** | **Std** | **Max** | | **Min** | |
| April | 0.0017 | 0.0020 | 0.0085 | | 2.0000e-008 | |
| May | 9.6375e-004 | 6.6980e-004 | 0.0039 | | 2.0000e-008 | |
| June | 0.0018 | 0.0019 | 0.0231 | | 2.0000e-008 | |
| July | 0.0021 | 0.0011 | 0.0062 | | 2.0000e-008 | |
| August | 0.0031 | 0.0012 | 0.0075 | | 2.0000e-008 | |
| September | 0.0027 | 0.0012 | 0.0083 | | 2.0000e-008 | |
| October | 0.0028 | 0.0015 | 0.0142 | | 2.0000e-008 | |
| November | 0.0030 | 0.0011 | 0.0074 | | 2.0000e-008 | |
| December | 0.0033 | 0.0013 | 0.0083 | | 2.0000e-008 | |
| Month | **p distance** | | | | | |
|  | **Average** | **Std** | | **Max** | | **Min** |
| April | 8.1386e-004 | 7.4692e-004 | | 0.0040 | | 0 |
| May | 8.6469e-004 | 7.6827e-004 | | 0.0040 | | 0 |
| June | 0.0015 | 0.0018 | | 0.0210 | | 0 |
| July | 0.0020 | 0.0011 | | 0.0060 | | 0 |
| August | 0.0030 | 0.0012 | | 0.0060 | | 0 |
| September | 0.0027 | 0.0013 | | 0.0080 | | 0 |
| October | 0.0024 | 0.0015 | | 0.0130 | | 0 |
| November | 0.0029 | 0.0011 | | 0.0070 | | 0 |
| December | 0.0032 | 0.0014 | | 0.0080 | | 0 |

**Table S3.** Patristic distance and p distance for PB1 genes of 2009 pandemic H1N1 influenza A viruses from April of 2009 to December of 2009.

| Month | **patristic distance** | | | | | |
| --- | --- | --- | --- | --- | --- | --- |
|  | **Average** | **Std** | **Max** | | **Min** | |
| April | 0.0014 | 0.0020 | 0.0085 | | 2.0000e-008 | |
| May | 8.9904e-004 | 7.4765e-004 | 0.0049 | | 2.0000e-008 | |
| June | 0.0014 | 0.0010 | 0.0084 | | 2.0000e-008 | |
| July | 0.0025 | 0.0020 | 0.0132 | | 2.0000e-008 | |
| August | 0.0030 | 0.0014 | 0.0068 | | 2.0000e-008 | |
| September | 0.0026 | 0.0011 | 0.0070 | | 2.0000e-008 | |
| October | 0.0032 | 0.0015 | 0.0088 | | 2.0000e-008 | |
| November | 0.0037 | 0.0015 | 0.0091 | | 2.0000e-008 | |
| December | 0.0040 | 0.0017 | 0.0101 | | 2.0000e-008 | |
| Month | **p distance** | | | | | |
|  | **Average** | **Std** | | **Max** | | **Min** |
| April | 6.6292e-004 | 6.5921e-004 | | 0.0040 | | 0 |
| May | 7.9491e-004 | 8.0073e-004 | | 0.0050 | | 0 |
| June | 0.0011 | 8.6979e-004 | | 0.0050 | | 0 |
| July | 0.0022 | 0.0018 | | 0.0120 | | 0 |
| August | 0.0029 | 0.0013 | | 0.0070 | | 0 |
| September | 0.0026 | 0.0012 | | 0.0070 | | 0 |
| October | 0.0031 | 0.0015 | | 0.0090 | | 0 |
| November | 0.0033 | 0.0014 | | 0.0080 | | 0 |
| December | 0.0037 | 0.0016 | | 0.0100 | | 0 |

**Table S4.** Patristic distance and p distance for PA genes of 2009 pandemic H1N1 influenza A viruses from April of 2009 to December of 2009.

| Month | **patristic distance** | | | | | |
| --- | --- | --- | --- | --- | --- | --- |
|  | **Average** | **Std** | **Max** | | **Min** | |
| April | 6.5632e-004 | 6.0486e-004 | 0.0037 | | 2.0000e-008 | |
| May | 9.0609e-004 | 7.3316e-004 | 0.0056 | | 2.0000e-008 | |
| June | 9.5554e-004 | 8.0812e-004 | 0.0051 | | 2.0000e-008 | |
| July | 0.0015 | 7.7759e-004 | 0.0042 | | 2.0000e-008 | |
| August | 0.0023 | 0.0011 | 0.0065 | | 2.0000e-008 | |
| September | 0.0023 | 0.0011 | 0.0065 | | 2.0000e-008 | |
| October | 0.0031 | 0.0011 | 0.0078 | | 2.0000e-008 | |
| November | 0.0030 | 0.0012 | 0.0082 | | 2.0000e-008 | |
| December | 0.0036 | 0.0013 | 0.0087 | | 2.0000e-008 | |
| Month | **p distance** | | | | | |
|  | **Average** | **Std** | | **Max** | | **Min** |
| April | 5.4553e-004 | 6.3592e-004 | | 0.0030 | | 0 |
| May | 8.4065e-004 | 7.9564e-004 | | 0.0060 | | 0 |
| June | 8.9977e-004 | 8.7530e-004 | | 0.0050 | | 0 |
| July | 0.0014 | 8.4985e-004 | | 0.0040 | | 0 |
| August | 0.0023 | 0.0011 | | 0.0060 | | 0 |
| September | 0.0023 | 0.0011 | | 0.0070 | | 0 |
| October | 0.0029 | 0.0011 | | 0.0070 | | 0 |
| November | 0.0029 | 0.0013 | | 0.0080 | | 0 |
| December | 0.0036 | 0.0013 | | 0.0080 | | 0 |

**Table S5.** Patristic distance and p distance for NA genes of 2009 pandemic H1N1 influenza A viruses from April of 2009 to December of 2009.

| Month | **patristic distance** | | | | | |
| --- | --- | --- | --- | --- | --- | --- |
|  | **Average** | **Std** | **Max** | | **Min** | |
| April | 0.0015 | 0.0011 | 0.0057 | | 2.0000e-008 | |
| May | 0.0018 | 0.0013 | 0.0063 | | 2.0000e-008 | |
| June | 0.0015 | 0.0012 | 0.0077 | | 2.0000e-008 | |
| July | 0.0014 | 0.0010 | 0.0050 | | 2.0000e-008 | |
| August | 0.0019 | 9.2074e-004 | 0.0043 | | 2.0000e-008 | |
| September | 0.0026 | 0.0017 | 0.0113 | | 2.0000e-008 | |
| October | 0.0026 | 0.0016 | 0.0099 | | 2.0000e-008 | |
| November | 0.0027 | 0.0014 | 0.0084 | | 2.0000e-008 | |
| December | 0.0032 | 0.0015 | 0.0092 | | 2.0000e-008 | |
| Month | **p distance** | | | | | |
|  | **Average** | **Std** | | **Max** | | **Min** |
| April | 0.0014 | 0.0011 | | 0.0060 | | 0 |
| May | 0.0019 | 0.0013 | | 0.0070 | | 0 |
| June | 0.0012 | 0.0012 | | 0.0080 | | 0 |
| July | 0.0014 | 0.0010 | | 0.0050 | | 0 |
| August | 0.0019 | 9.3654e-004 | | 0.0040 | | 0 |
| September | 0.0026 | 0.0018 | | 0.0120 | | 0 |
| October | 0.0024 | 0.0015 | | 0.0100 | | 0 |
| November | 0.0027 | 0.0015 | | 0.0080 | | 0 |
| December | 0.0030 | 0.0016 | | 0.0100 | | 0 |

**Table S6.** Patristic distance and p distance for NP genes of 2009 pandemic H1N1 influenza A viruses from April of 2009 to December of 2009.

| Month | **patristic distance** | | | | | |
| --- | --- | --- | --- | --- | --- | --- |
|  | **Average** | **Std** | **Max** | | **Min** | |
| April | 0.0019 | 0.0015 | 0.0080 | | 2.0000e-008 | |
| May | 0.0020 | 0.0014 | 0.0066 | | 2.0000e-008 | |
| June | 0.0016 | 0.0011 | 0.0067 | | 2.0000e-008 | |
| July | 0.0022 | 0.0030 | 0.0206 | | 2.0000e-008 | |
| August | 0.0020 | 0.0012 | 0.0066 | | 2.0000e-008 | |
| September | 0.0022 | 0.0013 | 0.0086 | | 2.0000e-008 | |
| October | 0.0028 | 0.0015 | 0.0086 | | 2.0000e-008 | |
| November | 0.0032 | 0.0016 | 0.0098 | | 2.0000e-008 | |
| December | 0.0030 | 0.0017 | 0.0099 | | 2.0000e-008 | |
| Month | **p distance** | | | | | |
|  | **Average** | **Std** | | **Max** | | **Min** |
| April | 0.0019 | 0.0014 | | 0.0060 | | 0 |
| May | 0.0017 | 0.0012 | | 0.0060 | | 0 |
| June | 0.0015 | 0.0010 | | 0.0070 | | 0 |
| July | 0.0020 | 0.0026 | | 0.0190 | | 0 |
| August | 0.0021 | 0.0013 | | 0.0070 | | 0 |
| September | 0.0022 | 0.0012 | | 0.0080 | | 0 |
| October | 0.0029 | 0.0015 | | 0.0080 | | 0 |
| November | 0.0031 | 0.0016 | | 0.0090 | | 0 |
| December | 0.0032 | 0.0017 | | 0.0100 | | 0 |

**Table S7.** Patristic distance and p distance for MP genes of 2009 pandemic H1N1 influenza A viruses from April of 2009 to December of 2009.

| Month | **patristic distance** | | | | | |
| --- | --- | --- | --- | --- | --- | --- |
|  | **Average** | **Std** | **Max** | | **Min** | |
| April | 0.0018 | 0.0015 | 0.0061 | | 2.0000e-008 | |
| May | 0.0021 | 0.0015 | 0.0081 | | 2.0000e-008 | |
| June | 0.0025 | 0.0018 | 0.0111 | | 2.0000e-008 | |
| July | 0.0019 | 0.0017 | 0.0092 | | 2.0000e-008 | |
| August | 0.0024 | 0.0021 | 0.0092 | | 2.0000e-008 | |
| September | 0.0023 | 0.0015 | 0.0082 | | 2.0000e-008 | |
| October | 0.0017 | 0.0016 | 0.0104 | | 2.0000e-008 | |
| November | 0.0018 | 0.0015 | 0.0101 | | 2.0000e-008 | |
| December | 0.0022 | 0.0019 | 0.0134 | | 2.0000e-008 | |
| Month | **p distance** | | | | | |
|  | **Average** | **Std** | | **Max** | | **Min** |
| April | 0.0015 | 0.0012 | | 0.0050 | | 0 |
| May | 0.002 | 0.001615 | | 0.009 | | 0 |
| June | 0.0020 | 0.0014 | | 0.0090 | | 0 |
| July | 0.0019 | 0.0017 | | 0.0090 | | 0 |
| August | 0.0023 | 0.0021 | | 0.0090 | | 0 |
| September | 0.0022 | 0.0015 | | 0.0070 | | 0 |
| October | 0.0014 | 0.0014 | | 0.0090 | | 0 |
| November | 0.0017 | 0.0015 | | 0.0100 | | 0 |
| December | 0.0020 | 0.0018 | | 0.0130 | | 0 |

**Table S8.** Patristic distance and p distance for NS genes of 2009 pandemic H1N1 influenza A viruses from April of 2009 to December of 2009.

| Month | **patristic distance** | | | | | |
| --- | --- | --- | --- | --- | --- | --- |
|  | **Average** | **Std** | **Max** | | **Min** | |
| April | 0.0017 | 0.0015 | 0.0089 | | 2.0000e-008 | |
| May | 0.0013 | 0.0012 | 0.0107 | | 2.0000e-008 | |
| June | 0.0026 | 0.0017 | 0.0117 | | 2.0000e-008 | |
| July | 0.0027 | 0.0016 | 0.0095 | | 2.0000e-008 | |
| August | 0.0036 | 0.0018 | 0.0077 | | 2.0000e-008 | |
| September | 0.0032 | 0.0021 | 0.0107 | | 2.0000e-008 | |
| October | 0.0037 | 0.0024 | 0.0166 | | 2.0000e-008 | |
| November | 0.0031 | 0.0021 | 0.0151 | | 2.0000e-008 | |
| December | 0.0033 | 0.0018 | 0.0106 | | 2.0000e-008 | |
| Month | **p distance** | | | | | |
|  | **Average** | **Std** | | **Max** | | **Min** |
| April | 0.0014 | 0.0013 | | 0.0070 | | 0 |
| May | 0.0011 | 9.6642e-004 | | 0.0050 | | 0 |
| June | 0.0011 | 0.0012 | | 0.0090 | | 0 |
| July | 0.0017 | 0.0014 | | 0.0080 | | 0 |
| August | 0.0028 | 0.0016 | | 0.0070 | | 0 |
| September | 0.0027 | 0.0019 | | 0.0100 | | 0 |
| October | 0.0029 | 0.0020 | | 0.0100 | | 0 |
| November | 0.0029 | 0.0020 | | 0.0130 | | 0 |
| December | 0.0033 | 0.0019 | | 0.0100 | | 0 |

**Table S9.** The parameters used in Model 1.

| Parameter | Description | Value |
| --- | --- | --- |
| 1/α | Duration of latency | 1 day |
| β_0_ | Mean transmission rate | 0.75 per day |
| β_1_ | Cyclic transmission rate | 0.3 per day |
| 1/*v* | Duration of infectiousness | 3 days |
| *T* | Days |  |
| *S*(0) | Initial fraction of susceptible individuals | 0.794 |
| *E(0)* | Initial fraction of exposed individuals | 0 |
| *I(0)* | Initial fraction of infected individuals | 0.006 |
| *R(0)* | Initial fraction of removed individuals | 0.2 |

**Table S10.** The parameters used in Model 2.

| Parameter | Description | Value |
| --- | --- | --- |
| 1/α | Duration of latency | 1 day |
| β_0_ | Initial transmission rate | 0.63 per day |
| 1/*v* | Duration of infectiousness | 3 days |
| *T* | Days |  |
| *S*(0) | Initial fraction of susceptible individuals | 0.857 |
| *E(0)* | Initial fraction of exposed individuals | 0.002 |
| *I(0)* | Initial fraction of infected individuals | 0.004 |
| *R(0)* | Initial fraction of removed individuals | 0.137 |

**Table S11**. The parameters used in Model 3.

| Parameter | Description | Value |
| --- | --- | --- |
| 1/α | Duration of latency | 1 day |
| β_1_ | Transmission rate for sub-population 1 | 2.1 per day |
| β_2_ | Transmission rate for sub-population 2 | 0.565 per day |
| β_3_ | Transmission rate between sub-populations | 0.01 per day |
| 1/*v* | Duration of infectiousness | 3 days |
|  | Fraction of population in sub-population 1 | 0.22 |
| *T* | Days |  |
| *S_1_*(0) | Initial fraction of susceptible individuals in sub-population 1 | 0.214 |
| *E_1_(0), E_2_(0)* | Initial fraction of exposed individuals in sub-populations 1 and 2 | 0 |
| *I_1_(0)* | Initial fraction of infected individuals in sub-population 1 | 0.006 |
| *R_1_(0), R_2_(0)* | Initial fraction of removed individuals in sub-population 1 and 2 | 0 |
| *S_2_(0)* | Initial fraction of susceptible individuals in sub-population 2 | 0.7797 |
| *I_2_(0)* | Initial fraction of infected individuals in sub-population 2 (at time t = 240) | 0.0003 |

**Table S12.** The parameters used in Model 4.

| Parameter | Description | Value |
| --- | --- | --- |
| 1/α | Duration of latency | 1 day |
| β | Transmission rate | 0.9 per day |
| 1/*v* | Duration of infectiousness | 3 days |
| *𝛾* | Scale of *d(t)* | 0.08, 0.09, or 0.11 per day |
| *d*(*t*) | Genetic distance function (normalized) |  |
| *T* | Days |  |
| *S*(0) | Initial fraction of susceptible individuals | 0.444 |
| *E(0)* | Initial fraction of exposed individuals | 0 |
| *I(0)* | Initial fraction of infected individuals | 0.006 |
| *R(0)* | Initial fraction of removed individuals | 0.2 |
| *N(0)* | Initial fraction of reserve non-susceptible individuals | 0.35 |

**Table S13.** The parameters used in Model 5.

| Parameter | Description | Value |
| --- | --- | --- |
| 1/α | Duration of latency | 1 day |
| β | Transmission rate | 0.63 per day |
| 1/*v* | Duration of infectiousness | 3 days |
| 1/φ | Duration of immunity | 110 days |
| *T* | Days |  |
| *S*(0) | Initial fraction of susceptible individuals | 0.794 |
| *E(0)* | Initial fraction of exposed individuals | 0 |
| *I(0)* | Initial fraction of infected individuals | 0.006 |
| *R(0)* | Initial fraction of removed individuals | 0.2 |


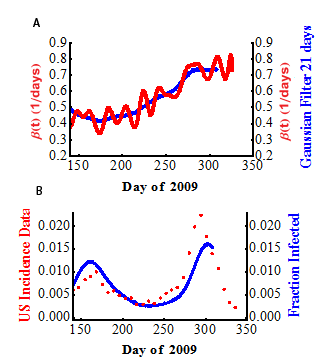


**Figure S1.** The oscillations of β_t_ seem essential to reproduce the two waves. (A) The filtered transmission function using a 21-day Gaussian filter, compared with the unfiltered transmission function with β_0_=0.63. (B) The reproduced disease prevalence generated by Model 1 using the filtered transmission function, compared with CDC data scaled for underreporting.
